# The effects of milk thistle on hepatic fibrosis due to methotrexate in rat

**Published:** 2011-06-01

**Authors:** Ali Reza Ghaffari, Hamid Noshad, Ali Ostadi, Morteza Ghojazadeh, Parviz Asadi

**Affiliations:** 1 Department of Internal Medicine, Sina hospital, Tabriz University of Medical Sciences, Liver and Gastrointestinal Research Center, Tabriz, IR Iran; 2 Department of Internal Medicine, Sina Hospital, Tabriz University of Medical Sciences, Tabriz, IR Iran; 3 Department of Physiology, Tabriz University of Medical Sciences, Tabriz, IR Iran

**Keywords:** Milk thistle, Silybum marianum, Methotrexate, Drug toxicity, Liver, Rats

## Abstract

**Background:**

Extracts of milk thistle (MT), Silybum marianum, have been used as medical remedies since the time of ancient Greece. Methotrexate is a potentially hepatotxic drug.

**Objectives:**

To clarify the hepatoprotective effects of MT on methotrexate.

**Materials and Methods:**

From January 2010 to April 2010, 30 male rats were recruited into three 10-rat subgroups in Tabriz University of Medical Sciences. Normal saline was injected intraperitoneally in the first group (A; the controls); intraperitoneal methotrexate plus oral MT extract were administered to the second group (B) and intraperitoneal methotrexate alone was given to the third group (C). Pre- and post-interventional measuring of serum parameters were carried out every 15 days. After six weeks, the rats were decapitated and histopathological evaluation of liver was done.

**Results:**

Serum liver enzymes (AST, ALT), alkaline phosphatase, total and direct bilirubin, creatinine and BUN were measured on days 0, 15, 30, 45. They were significantly higher in the group C, comparing with other two groups. Serum albumin was the least in group C animals as well. There were no significant differences between groups A and B. The mean±SD fibrosis score using semi-quantitative scoring system (SSS) was 1.25±0.46, 1.40±0.52 and 6.70±0.82, in groups A, B and C, respectively (p<0.001).

**Conclusions:**

MT extract can effectively prevent methotrexate-induced liver dysfunction and fibrosis in rats.

## 1. Background

Methotrexate (MTX) is a potent hepatotoxic agent. This drug is effective in various cancers and immunologic disorders. It is used frequently in rheumatoid arthritis and psoriasis. This drug when used without follow-up has many side effects like hepatotoxicity and bone marrow suppression. MTX is accumulated in liver and is hepatotoxic. It seems that folic acid can reduce MTX side effects but it is not completely clarified. Clinicians use the drug frequently, so they would like to reduce its side effects especially its hepatotoxic effects [1]. It was shown that milk thistle (Silybum marianum) has beneficial effects on hepatotoxicity [2]. The objective of this study was to clarify the effect of MT on MTX-induced hepatotoicity in an animal model. In animal models, it has been shown that MT prevents atherosclerotic plaque formation in aorta. It has been shown that the cisplatin and cyclosporine side effects reduced when MT was administered in mice [3][4]. Reports showed that silymarin promote DNA polymerase, stabilize all membranes, inhibits free radicals and increases glutathione concentration, so it could protect liver from hepatotoxic agents. Silibinin is able to stimulate the activity of the DNA-dependent RNA polymerase I and causes an increase in rRNA synthesis. It accelerates formation of intact rRNA polymerase with resultant formation of new hepatocytes [5]. Silymarin inhibits lipoxygenase cycle, leukotrienes and free radicals formation in mice Kupffer cells, so inflammation may be reduced [6]. Treatment with MT has been usual since 2000 years ago and it is mentioned as a hepatoprotective agent [7]. MT is found in many areas all around the world and is cultured in North and South parts of Iran. This drug is absorbed via the gastrointestinal tract; the maximum blood level is reached after 2-4 hours. The half-life of the drug is six hours. About 80% of MT is secreted into the bile and its bioavailability depends on its formulation [8]. Sylibin is the most effective agents in MT and is known as an antioxidant and hepatoprotective agent. Its concentration in bile is 60 times greater than the blood. Silymarin has various cardiovascular effects [9]. Silymarin inhibits liver enzymes like gamaglutamil transpeptidase (GGT), alanine transaminase (ALT) and aspartate transaminase (AST) in rats [5] This drug blocks hepatic fibrosis due to biliary obstruction in mice. In one study a formulation of silymarin extract (Legalon) was used in 2637 patients with chronic liver disease for eight weeks when the liver enzymes remarkably decreased in 88% of patients. Side effects were seen in lesser than 1% of patients [10]. Silymarin is widely used in poisoning with Amanita fungus and reduces mortality significantly (60%-80%). The effects of silymarin on alcoholic liver damages are controversial, but in a controlled double blind clinical trial this drug could improve liver enzyme level and histopathologic liver features after four weeks in alcoholic hepatotoxicity [11]. In one study, silymarin could reduce mortality in patients with alcoholic cirrhosis after four years. In another study, however, silymarin could not reduce hepatic mortality in cirrhotic patients [12]. Silymarin effects on hepatic damages due to hepatitis are also controversial. In a double blind clinical trial, 20 patients with chronic active hepatitis received 240 mg of silybin complex (silipide) two times a day for seven days; the GGT level reduced significantly [13]. In another study, 29 patients with viral hepatitis treated with silymarin and 28 patients received placebo; serum bilirubin, AST and ALT levels significantly reduced in the treatment group but in another study with 151 patients with viral hepatitis this drug could not improve their clinical condition [14]. Silymarin has other therapeutic effects. It reduces LDL cholesterol level and atherosclerotic plaque formation in rabbit and mice [15]. This agent has some mild side effects like allergic reactions in sensitive patients. In animal models, silymarin in higher doses has not any side effects. Long-term use of this drug was safe. Silymarin also is safe in pregnancy, lactation and children [12].

## 2. Objectives

This study tries to determine the hepatoprotective effects of MT on MTX-induced hepatotoxicity.

## 2. Materials And Methods

In an experimental study, 30 rats (weigh 250-300 g) were used. The rats were in animal house for one week and had access to water and food ad libitum. Temperature was kept at 37 °C. After one week, the rats were randomly divided into three equal groups: Group A rats received normal saline (600 mg/kg); group B received MTX (100 μg/kg) intraperitoneally and silymarin (600 mg/kg) orally; group C rats received MTX (100 μg/kg) intraperitoneally alone. We used 1 mL of MTX (1000 mg/10 mL) and diluted it in 99 mL of normal saline and then 1 mL of product was diluted in 9 mL of normal saline (100 µg/mL MTX). It was injected by insulin syringes. MT was administered orally by a special syringe for rats after four hours. This study was done in Education Development Center of Tabriz University of Medical Sciences, Tabriz, Iran. The study was conducted under supervision of a zoologist and pharmacist, professional in these kinds of animal studies.

MT seeds were obtained from different areas of Aras river in East Azarbayjan, North-West of Iran. In pharmacognosy laboratory of pharmacy faculty, dry seeds were milled and processed with hexane; the silymarin and its flavonolignan were extracted using succilating method. After solvent evaporation, the amount of the total flavonolignans of silymarin was measured by spectrophotometry. Extracted materials were fractionated, using SPE (sep-pak) cartridges and methanol-water mixture. Solvent of the obtained fractions was evaporated and the product was used in this study. Tabriz University of Medical Sciences Ethical Committee approved this study. All animals received humane care according to the criteria outlined in the "Guide for the Care and Use of Laboratory Animals" prepared by the National Academy of Sciences and published by the National Institutes of Health (NIH publication 86-23 revised 1985). For biochemical studies, blood samples were taken before the intervention and 15, 30 and 45 days post-intervention. Serum samples were examined for alanine aminotransferase (ALT), aspartate aminotransferase (AST), alkalin phosphatase (ALP), blood urea nitrogen (BUN), creatinine (Cr), bilirubin (Bil), and albumin (Alb). Every morning (8-9 am), MTX was injected; MT was given orally by special syringe four hours later. Six weeks after intervention, rats were killed, their livers were fixed in 10% formalin and studied for histopathologic changes. The samples were scored using a semi-quantitative scoring system (SSS) [16] as below:

< 2: normal;

2-6: mild fibrosis;

6-10: moderate fibrosis;

> 10: sever fibrosis;

The data were analyzed by SPSS® for Windows® ver 17. The continuous variables were first tested to see if they were distributed normally. Descriptive statistics, including the mean and standard deviation (SD) was calculated for all variables. One-way ANOVA or Kruskal-Wallis test was used to compare means of three or more groups. Pairwise comparisons of the study groups were performed using Tukey's HSD and Mann-Whitney U test as post hoc tests. A p < 0.05 was considered statistically significant.

## 3. Results

Biochemical parameters were measured before the intervention, and 15, 30, and 45 days after the intervention. The creatinine level was significantly different among all the studied groups (p < 0.001) but groups A and B. Therefore, creatinine level was lower in patients treated with MT (Table 1). BUN was also significantly different among the studied groups (p < 0.001). BUN level was better in rats treated with MT (Table 1). ALT was also significantly different among all (p < 0.001) but groups A and B (p = 0.211). ALT level was lower in group B (Table 1). AST level was also significantly different among all studied groups (p < 0.001) (Table 1). Total bilirubin level was significantly different among all (p < 0.001) but groups A and B was not remarkable. Total bilirubin level was lower in rats treated with MT (Table 1). Direct bilirubin level was significantly different among all studied groups (p < 0.001) (Table 1).

**Table 1:** Mean±SD of measured biochemical parameters in the studied groups

**Group**	**Cr **(mg/dL)	**BUN **(mg/dL)	**ALT **(IU/L)	**AST **(IU/L)	**BIL **(mg/dL)	**BILD **(mg/dL)	**Alb **(g/dL)	**ALP **(IU/L)
**Group A**								
**1 (Day 0)**	0.56 ± 0.06	11.33 ± 1.12	32.40 ± 7.38	119.70 ± 69.91	0.56 ± 0.86	0.40 ± 0.70	3.79 ± 025	120.00 ± 4366
**2 (Day 15)**	0.56 ± 0.5 0.54	11.40 ± 1.17	31.70 ± 6.18	99.60 ± 9.78	0.28 ± 0.08	0.14 ± 0.03	3.91 ± 0.26	110.80±34.10
**3 (Day 30)**	± 0.5 0.54 ±	11.50 ± 1.26	32.40 ± 7.38	98.60 ± 8.30	0.28 ± 0.08	0.14 ± 0.04	3.79 ± 0.25	110.10±33.87
**4(Day 45)**	0.08	11.11 ± 1.00	33.30 ± 3.23	95.70 ± 8.43	0.27 ± 0.12	0.14 ± 0.02	3.73 ± 0.20	99.60 ± 7.42
**Group B**								
**1 (Day 0)**	0.55 ± 005	11.41 ± 1.16	32.40 ± 7.38	111.00 ± 52.37	0.28 ± 010	0.15 ± 0.02	3.79 ± 0.25	105.10 ± 1513
**2 (Day 15)**	0.57 ± 0.05	11.72 ± 1.02	34.00 ± 4.16	86.80 ± 8.01	0.30 ± 0.11	0.13 ± 0.03	3.79 ± 0.25	126.90 ± 40.42
**3 (Day 30)**	0.56 ± 0.05	11.52 ± 1.15	34.00 ± 4.16	85.50 ± 5.23	0.31 ± 0.07	0.14 ± 0.03	3.79 ± 0.25	117.30 ± 31.61
**4(Day 45)**	0.57 ± 0.05	11.41 ± 1.16	34.90 ± 4.40	85.60 ± 4.81	0.27 ± 0.09	0.14 ± 0.02	3.79 ± 0.25	105.30 ± 13.87
**Group C**								
**1 (Day 0)**	0.56 ± 005	11.52 ± 1.15	32.40 ± 7.38	114.80 ± 50.63	0.56 ± 086	0.42 ± 0.74	3.79 ± 025	129.40 ± 6224
**2 (Day 15)**	0.60 ± 0.04	12.41 ± 1.94	75.60 ± 17.04	187.80 ± 37.54	1.58 ± 0.27	1.23 ± 0.27	3.69 ± 0.22	128.00 ± 14.17
**3 (Day 30)**	0.63 ± 0.07	16.50 ± 1.43	183.30 ± 43.46	362.90 ± 57.51	2.31 ± 0.64	1.88 ± 0.68	3.05 ± 0.21	139.00 ± 15.65
**4(Day 45)**	0.72 ± 0.10	16.90 ± 1.91	596.00 ± 101.21	800.70 ± 86.99	2.91 ± 0.63	2.53 ± 0.58	2.80 ± 0.21	147.60 ± 24.67
**P value**	< 0.001	< 0.001	< 0.001	< 0.001	< 0.001	< 0.001	< 0.001	0.01

**Figure 1:**
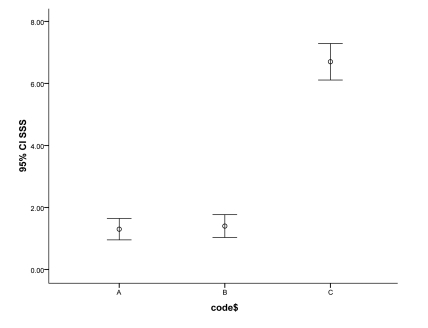
Error bar chart of SSS scores at the end of the study.

Albumin level was significantly different among the studied groups (p < 0.001) (Table 1). Bilirubin was lower and albumin was higher in group B rats. ALP was also significant among the studied groups (p = 0.01). The difference between groups A and C was significant (p = 0.01) but it was not among other paired groups (Table 1). It must be emphasized that we did not measure prothrombin time. The mean ± SD SSS score was 1.25 ± 0.49 in group A, 1.40 ± 0.52 in group B and 6.70 ± 0.82 in group C rats. The score was significantly higher in group C (p < 0.001)-fibrosis was more severe in rats exposed to MTX without MT extract. The mean SSS score was significantly different among the studied groups. Difference between paired groups was only significant between groups A and C (p < 0.001) and also groups B and C (p < 0.001) (Figure 1).

## 5. Discussion

In this study protective effects of MT on MTX-induced liver damage in rats was investigated. We found that the mean levels of ALT, AST, ALP and bilirubin in rats that received MTX plus MT were significantly lower than those animals received only MTX. Difference of studied parameters between MTX plus MT and control group (that received only normal saline) was not significant. Up to now, various studies revealed protective effects of MT in hepatic damages [17]. In these studies, it was shown that extract of MT reduced treatment period of acute and chronic hepatitis [18]. Protective effects of MT in fatty liver, cirrhosis, viral hepatitis, ischemic liver damage and cancer was shown previously [19]. In our study, also SSS score in MTX plus MT group was significantly lower than that in MTX group which reflected protective effects of MT. Buzzelli and colleagues showed that administration of MT extract to 20 patients with chronic active hepatitis reduced serum liver enzymes, ALP and bilirubin levels after seven weeks [20]. In our study, liver enzymes and bilirubin were reduced in rats received MT. Giese showed that MT extract has antihepatitic effects[21]. Dhiman also emphasized protective effects of MT on hepatic disease [22]. Mayer revealed protective and curative effects of MT on viral hepatitis [23]. Rambaldi and colleagues showed these protective and curative effects in viral and alcoholic hepatitis [24]. Jacobs et al. emphasized the protective effects of MT extracts in hepatic disease [25]. Other investigators like Gassileth (2008), Ross (2008), Raina (2008), and Ramakrishnan (2008), also emphasized the efficacy of MT extract on liver disease and its safety [7][26][27][28]. Protective mechanisms of MT extract in liver disease are mentioned in different studies [29][30][31]. Some of them are antioxidant, anti- lipid peroxidase, anti-fibrinolytic, anti-inflammatory and immunomodulatory effects, induction of cell formation, glutathione inhibition, reduction of leukotrienes, reduction of tumor promoters and P450 inhibition. We found that the mean BUN and creatinine level were lower in the group that received MT extract. So reno-protective effects are also proposed [32]. In animal studies, this drug prevented atherosclerotic plaque formation in aorta [33]. Previous studies revealed that silymarin prevents acetaminophen and tetrachloromethane hepatotoxic effects [22]. Reports showed that MT may promote DNA polymerase, stabilize all membrane, inhibit free radicals and increase glutathione concentration, so it protects liver against hepatotoxic agents [34]. Promotion of DNA polymerase leads to rRNA synthesis and hepatocellular regeneration. By increasing glutathione concentration, it stabilizes superoxide dismutase and glutathione peroxidase [18]. Different animal studies showed that silymarin protects hepatocytes against viruses, chemical agents, fungal toxins and alcohol-premedication. This drug protects animals against fatal effects of Amanita toxins [12][35]. Silymarin premedication prevents hepatotoxic effects of halothane, thallium tetrachloride and acetaminophen in animal studies [7]. Silymarin inhibits liver enzymes like gamma-glutamyl transpeptidase (GGT), ALT and AST in rats [36]. In one study, Silymarin could reduce mortality in patients with alcoholic cirrhosis after four years. On the other hand, in another study, silymarin could not reduce hepatic mortality in cirrhotic patients [35]. In our study it was shown that MT extract protects liver against MTX hepatotoxic effects. Liver enzymes (AST, ALT and ALP), bilirubin and albumin remain unchanged. Also MT extract prevents kidney injury due to MTX in rat. BUN and creatinine levels remain unchanged. MT extract significantly prevents liver damage due to MTX in rat, so similar human studies are required. Studies on the effect of MT extracts on drug-induced kidney injury are therefore warranted. Similar results would justify use of MT extract in patients receiving MTX as a prophylactic agent against hepatic side effects.
